# Immune Imprinting Identified in Phage-Display Antibody Libraries Derived from Early Wild-Type and Late Omicron COVID-19 Convalescents

**DOI:** 10.3390/v18010132

**Published:** 2026-01-20

**Authors:** Boyang Li, Mengxuan Wang, Fang Huang, Wei Wu, Jiaxin Fan, Lu Yang, Yongbing Pan, Mifang Liang, Kai Duan

**Affiliations:** 1 Hubei Provincial Vaccine Technology Innovation Center, State Key Laboratory of Novel Vaccines for Emerging Infectious Diseases, National Engineering Technology Research Center for Combined Vaccines, Wuhan Institute of Biological Products Co., Ltd., Wuhan 430207, China; by_li@foxmail.com (B.L.); 2 Wuhan Riverflow Ab Biotechnology Co., Ltd., Wuhan 430207, China; 3 Hubei Jiangxia Laboratory, Wuhan 430207, China

**Keywords:** SARS-CoV-2, Omicron, immune imprinting, original antigenic sin, phage display antibody library, broadly neutralizing antibody, non-neutralizing antibody, receptor-binding domain

## Abstract

The rapid evolution of SARS-CoV-2, particularly the emergence of Omicron subvariants, has significantly reduced the efficacy of existing vaccines and monoclonal antibodies. This study investigates the phenomenon of immune imprinting by comparing two phage display antibody libraries derived from early 2020 wild-type SARS-CoV-2 convalescents (WT-AbLib) and early 2023 Omicron convalescents (Omi-AbLib). The capacity and diversity of both antibody libraries were systematically evaluated. The libraries were screened using BF.7 and XBB.1.5 antigens. WT-AbLib showed markedly reduced diversity after Omicron antigen selection, with dominant clones shifting from IGHV3-66-class broadly neutralizing antibodies (bnAbs) targeting the receptor-binding motif to IGHV1-46-class broadly non-neutralizing antibodies targeting conserved lateral receptor-binding domain (RBD) sites. Omi-AbLib maintained higher diversity, but dominant antibodies were also non-neutralizing and targeted the same conserved lateral region. These findings suggest that immune imprinting drives the dominance of broadly non-neutralizing antibodies following Omicron breakthrough or reinfection. This phenomenon provides a mechanistic explanation for persistent viral evasion and recurrent infection, and highlights major challenges for the development of next-generation broadly neutralizing therapeutics.

## 1. Introduction

Since its initial outbreak in late 2019, SARS-CoV-2 has undergone rapid evolution, with the Omicron variant, emerging in late 2021, distinguished by high transmissibility and extensive Spike protein mutations [1,2]. These changes have driven immune evasion, substantially reducing the effectiveness of vaccines and monoclonal antibody therapies designed against the wild-type (WT) strain [3,4,5,6,7]. This viral evolution has spotlighted immune imprinting, in which initial WT exposure shapes subsequent antibody responses, often biasing the repertoire toward cross-reactive, non-neutralizing antibodies via memory B cell recall, thus limiting broadly neutralizing antibody (bnAb) generation against new variants [8,9,10]. Understanding these immune dynamics is crucial for developing durable therapeutic strategies against evolving strains.

Prior studies, such as those by Cao et al., have used single-cell sequencing and deep mutational scanning to show that WT-induced antibodies target conserved, non-neutralizing receptor-binding domain (RBD) epitopes under Omicron pressure, compromising neutralization [8,11]. However, these in vivo profiling approaches lack systematic analysis of antibody repertoire dynamics during variant-specific selection. Phage-display technology offers a complementary and controllable platform, simulating immune selection in vitro to quantify shifts in antibody diversity and specificity.

Here, we investigate immune imprinting by comparing phage-display antibody libraries from WT-infected convalescents in early 2020 (WT-AbLib) and Omicron-infected convalescents in early 2023 (Omi-AbLib). We screened the libraries against BF.7 (BA.5.2.1.7) and XBB.1.5 antigens. BF.7 is a sublineage of the Omicron BA.5 branch and represents a further evolutionary step of BA.5, carrying key RBD mutations such as R346T that map to major neutralizing antibody epitopes. Immunologically, BF.7 retains the high transmissibility of BA.5 while exhibiting enhanced immune evasion against antibodies elicited by prior infection or vaccination, and it was a predominant circulating strain during the winter of 2022 to early 2023. Therefore, BF.7 serves as a representative variant for evaluating antibody adaptation and bias under BA.5-background immune pressure, closely matching the immunological context of Omi-AbLib donors. In contrast, XBB.1.5 is a recombinant Omicron lineage derived from BA.2 sublineages and represents a more extreme evolutionary state. It simultaneously harbors extensive immune-evasive mutations and key RBD substitutions such as F486P, which enhance human angiotensin-converting enzyme 2 (hACE2) binding while maintaining strong immune escape. Functionally, XBB.1.5 represents a rare combination of high receptor affinity and profound immune evasion, making it an ideal antigen for probing the limits of antibody breadth and assessing whether pre-existing immune imprinting constrains responses to newly emerged epitopes.

By combining these antigenic probes, we assessed antibody library diversity and dominant antibody shifts, revealing a transition from broadly neutralizing antibodies targeting the receptor-binding motif (RBM) to broadly non-neutralizing antibodies targeting conserved lateral RBD sites. These findings provide novel insights into immune imprinting’s role in recurrent infections and informing the design of bnAbs and vaccines against emerging SARS-CoV-2 variants.

## 2. Materials and Methods

### 2.1. Cells, Phages, Proteins, Vectors, and Antibody Libraries

HEK293T and Huh7 cells were purchased from the American Type Culture Collection (ATCC, Manassas, VA, USA). Cells were cultured in Dulbecco’s Modified Eagle Medium (DMEM, Gibco, Grand Island, NY, USA) supplemented with 10% fetal bovine serum (FBS, Gibco, Grand Island, NY, USA) at 37 °C with 5% CO_2_. Cell passages were performed every two days using 0.05% Trypsin-EDTA (Gibco, Grand Island, NY, USA). XL1-Blue competent cells were purchased from Beijing Biomed Gene Technology Co., Ltd. (Beijing, China). M13KO7 helper phage was obtained from New England Biolabs (NEB, Ipswich, MA, USA). SARS-CoV-2 WT and various mutant strains’ RBD and spike proteins were purchased from Jiangsu East-Mab Biomedical Technology Co., Ltd. (Nantong, China); SARS-CoV-2 RBD protein fused to a mouse Fc tag was purchased from Nanjing Novozan Biotech Co., Ltd. (Nanjing, China). The pcDNA3.4 vector was from Nanjing GenScript Biotechnology Co., Ltd. (Nanjing, China), and the WT-AbLib and Omi-AbLib antibody libraries were provided by the National Institute for Viral Disease Control and Prevention, China CDC. WT-AbLib represents antibody repertoires associated with wild-type SARS-CoV-2 infection occurring in early 2020, a period before the initiation of COVID-19 vaccination programs. At that time, population exposure was largely limited to the ancestral strain, and vaccine-induced immune pressure was absent, thus reflecting antibody responses primarily shaped by primary infection. In contrast, Omi-AbLib was constructed in early 2023 and reflects antibody repertoires formed during the winter of 2022 to early 2023, when the Omicron sublineage BF.7 was the predominant circulating strain. By this stage of the pandemic, most individuals in the general population had completed two to three doses of inactivated SARS-CoV-2 vaccines prior to infection. Accordingly, antibodies represented in Omi-AbLib were shaped by immune backgrounds resulting from both vaccination and subsequent Omicron infection, rather than from primary viral exposure alone. These two antibody libraries therefore correspond to donor populations with distinct immunological histories, providing a basis for comparative analysis of antibody repertoire responses under different antigenic selection pressures.

### 2.2. Phage Library Screening

The construction and screening of the phage antibody library followed previously established methods [12,13]. During the enrichment process, purified SARS-CoV-2 WT and Omicron RBD and spike proteins were used as antigens. The screening steps adhered to the standard phage enrichment method described by Barbas and Burton (1996) [14,15]. Fab clones were sequenced by Beijing Tianyihuiyuan Biotechnology Co., Ltd. (Beijing, China) to obtain the variable region sequences of the heavy and light chains.

### 2.3. Antibody Sequence Alignment and Analysis

To evaluate the diversity and VJ pairing preferences of the phage library, multiple independent clones were randomly selected for Sanger sequencing after each round of library construction and screening. The sequencing data that met quality control standards were aligned using AbAlign software 1.2.11 [16]. The diversity of the library was assessed using diversity indices such as Normalized Shannon Diversity, Shannon Evenness, Gini-Simpson Diversity Index, and Simpson Evenness. VJ pairing analysis was performed to reveal the gene usage preferences of the antibodies.

### 2.4. Fab Expression

Fab clones were inoculated into 2×YT liquid medium and cultured at 37 °C with shaking at 250 rpm for 16 h. The culture was then transferred to fresh medium at a 1:100 dilution and further incubated for 3 h. IPTG was added to a final concentration of 1 mM to induce Fab expression, and the culture was incubated at 30 °C for 16 h. The culture was centrifuged at 4000 rpm to collect the supernatant, which was stored at −20 °C for future use.

### 2.5. ELISA

SARS-CoV-2 RBD or spike protein was coated onto microtiter plates at 1 μg/mL and incubated overnight at 4 °C. The plates were washed three times with PBST, and then blocked with 5% skimmed milk at 37 °C for 1 h. After washing three times with PBST, Fab supernatant or serially diluted IgG antibodies (starting concentration of 0.5 μg/mL) were added, and the plates were incubated at 37 °C for 1 h. After washing five times, HRP-conjugated secondary antibodies (anti-human Fab or IgG-Fc, Sigma-Aldrich, St. Louis, MO, USA) were added at a 1:5000 dilution, and incubated for 1 h at 37 °C. After five washes, TMB substrate was added for color development, and the optical density at 450 nm (OD_450_) was measured. Data were analyzed using GraphPad Prism 8 software, and binding curves were plotted to calculate the half-maximal effective concentration (EC_50_).

### 2.6. Antibody Expression and Purification

Heavy and light chain genes of the antibodies were codon-optimized and synthesized by Nanjing GenScript Biotechnology Co., Ltd. (Nanjing, China), and cloned into the pcDNA3.4 vector. Antibody expression plasmids were co-transfected into HEK-293T cells at a 1:1 ratio using X-tremeGENE™ HP DNA Transfection Reagent (Roche Diagnostics GmbH, Basel, Switzerland). The cells were cultured at 37 °C with 5% CO_2_ for 72 h, and the culture supernatant was collected. Antibodies were purified using Protein-G affinity chromatography columns (GE Healthcare, Chicago, IL, USA) and stored at −80 °C.

### 2.7. Pseudovirus Neutralization Assay

Fab supernatant or serially diluted IgG antibodies (starting concentration of 20 μg/mL) were incubated with SARS-CoV-2 pseudovirus (50 μL) from different strains at 37 °C for 1 h. Huh7 cells (2.5 × 10^4^ cells/100 μL/well) were added and incubated at 37 °C with 5% CO_2_ for 24 h. Luciferase activity was measured using the Bright-Lite Luciferase Assay System (Nanjing Novozan Biotech Co., Ltd., Nanjing, China) to evaluate the infection rate. Neutralization was determined by comparing with the virus control and cell control groups. Data were analyzed using GraphPad Prism 8 software, and inhibition curves were plotted to calculate the half-maximal inhibitory concentration (IC_50_).

### 2.8. RBD-ACE2 Binding Blocking Assay (FACS Method)

hACE2 expression plasmids were transfected into HEK293T cells and incubated for 24 h. SARS-CoV-2 RBD protein with a mouse Fc tag (2 μg/mL) was mixed with monoclonal antibodies or isotype control IgG (RV3, an anti-rabies virus monoclonal antibody) at a 1:10 molar ratio, and incubated at 4 °C for 1 h. The mixture was then incubated with hACE2-expressing HEK293T cells at 4 °C for 1 h. Cells were stained with anti-mouse IgG PE-conjugated antibody and anti-human IgG FITC-conjugated antibody (Sigma-Aldrich, St. Louis, MO, USA), and analyzed using a FACSMelody flow cytometer (BD, Franklin Lakes, NJ, USA). Data were analyzed using FlowJo software 10.8.1.

### 2.9. Structural Prediction

The binding model of antibodies with the RBD protein was predicted using AlphaFold3 (Google DeepMind) [17]. Heavy and light chain sequences of the antibody and the RBD protein sequence were input into the AlphaFold3 system, and 20 independent predictions were performed with a random seed to generate 100 different conformations. The models were scored using the InterfaceAnalyzer module in Rosetta (source release 351; RosettaCommons), and the optimal binding model was selected. Visualization and image processing were performed using PyMOL software 2.5.5 [18].

## 3. Results

### 3.1. Diversity Analysis and Quality Evaluation of Two Antibody Libraries Derived from WT and Omicron Patient Samples

**Figure 1:**
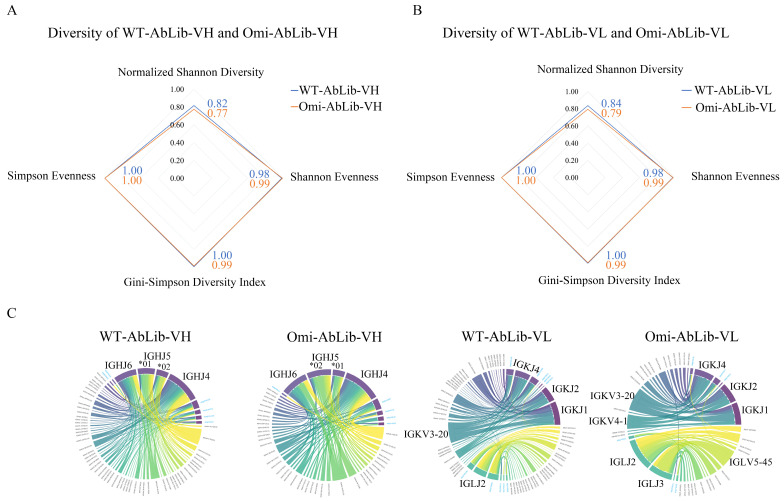
Diversity and VJ gene usage characteristics of WT-AbLib and Omi-AbLib. (**A**) Diversity metrics (Normalized Shannon Diversity, Simpson Evenness, Shannon Evenness, and Gini-Simpson Diversity Index) for WT-AbLib-VH and Omi-AbLib-VH. All four indices range from 0 to 1, with higher values indicating greater diversity and/or evenness of sequence distribution. (**B**) Diversity metrics of the VL repertoires in WT-AbLib and Omi-AbLib, calculated using the same indices as in (**A**). (**C**) Chord diagrams showing V–J gene family pairing patterns in the VH and VL regions of WT-AbLib and Omi-AbLib. The width of each ribbon reflects the relative abundance of a given V–J pairing, and values in parentheses indicate family-level frequencies. Colors represent distinct VH or VL gene families.

WT-AbLib and Omi-AbLib are phage display antibody libraries constructed from convalescents of SARS-CoV-2 WT and Omicron infections, respectively. Statistical analyses revealed that both libraries exhibited high diversity, with Simpson’s Evenness, the Gini-Simpson Diversity Index, and Shannon’s Evenness values approaching 1, and a normalized Shannon diversity of approximately 0.8 (Figure 1A,B), indicating a high level of internal diversity. Furthermore, chord diagrams demonstrated the extensive diversity of both heavy chain (VH) and light chain (VL) families, as well as a rich variety of VJ gene combinations (Figure 1C).

### 3.2. Screening Analysis of WT-AbLib and Omi-AbLib

**Figure 2:**
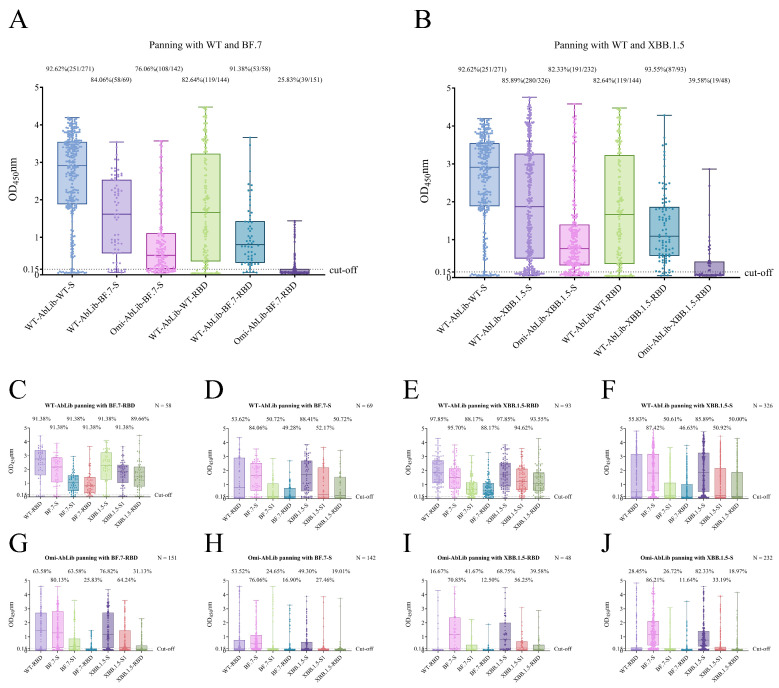
Panning efficiency and binding specificity of WT-AbLib and Omi-AbLib against SARS-CoV-2 RBD and S proteins from various variants. (**A**,**B**) Boxplots showing ELISA OD_450_ values of Fab clones derived from WT-AbLib and Omi-AbLib following panning with WT and BF.7 (**A**) or WT and XBB.1.5 (**B**) proteins. The dashed line indicates the positivity cutoff. The number and percentage of Fab clones exceeding the cutoff are annotated above each group. (**C**–**J**) Further ELISA analysis of positive Fab clones (OD_450_ > 2.0) selected from A and B, comparing their binding reactivity against WT-S1, BF.7, and XBB.1.5 RBD, S1, and trimeric S proteins. Each boxplot shows the distribution of OD_450_ values across multiple Fab clones, with the cut-off line indicating the threshold for positive binding. The number of tested Fab clones and the percentage of positive binders are indicated above each group.

The WT-AbLib and Omi-AbLib antibody libraries were panned with the RBD and S proteins of SARS-CoV-2 WT, BF.7, and XBB.1.5, followed by binding activity assessment. Significant differences in panning efficiency and antigen binding were observed between the two libraries. Using WT S protein as the reference antigen for WT-AbLib panning, the Fab-positive rate for BF.7 S protein enrichment decreased from 92.62% to 84.06%, while the Fab-positive rate in Omi-AbLib dropped further to 76.06%. The mean OD_450_ values exhibited a similar trend (Figure 2A,B).

To further evaluate the antigen specificity of the Fab-positive clones, we conducted ELISA assays using multiple antigens. Results showed that when panning WT-AbLib with BF.7 or XBB.1.5 RBD proteins, the positive rates of Fab binding to S, S1, and RBD proteins were similar, but the OD_450_ values decreased sequentially (Figure 2C,E). Similarly, when using BF.7 or XBB.1.5 S proteins for panning, the Fab-positive rates and OD_450_ values for S, S1, and RBD proteins also followed a descending trend (Figure 2D,F). In contrast to WT-AbLib, when panning Omi-AbLib with BF.7 RBD protein, the Fab-positive rate and mean OD_450_ value for WT RBD protein were higher than those of the other three groups, and a consistent decreasing trend was observed for the Fab-positive rate and OD_450_ values for S, S1, and RBD proteins (Figure 2G–J). Additionally, for the same antigen enrichment, the Fab-positive rate and OD_450_ values were consistently higher in WT-AbLib compared to Omi-AbLib (Figure 2C–J).

### 3.3. Sequence Analysis of Dominant Antibodies from Two Libraries

**Figure 3:**
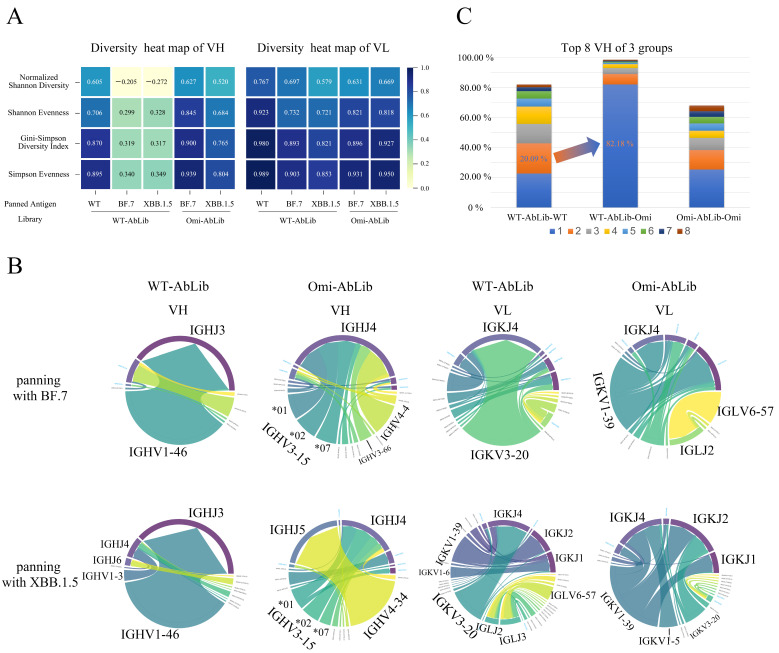
Repertoire reshaping after panning with SARS-CoV-2 antigens. (**A**) Heatmaps showing diversity metrics (normalized Shannon diversity, Shannon evenness, Gini-Simpson diversity index, and Simpson evenness) for VH and VL repertoires in WT-AbLib and Omi-AbLib panned with SARS-CoV-2 WT and Omicron. Higher values (closer to 1) indicate greater diversity and evenness in sequence distribution. (**B**) Chord diagrams illustrate the usage of VH (left) and VL (right) gene families in the WT-AbLib and Omi-AbLib libraries after panning. The top row shows results for BF.7 panning, while the bottom row corresponds to XBB.1.5. The length of each arc represents the relative abundance of the respective gene family, with each color denoting a unique VH or VL family. (**C**) Stacked bar chart showing the cumulative proportion of the top eight most frequent VH CDR3 sequences across three groups. Colors represent distinct VH CDR3 clonotypes. The arrow highlights a shared VH CDR3 clonotype detected in both WT-AbLib-WT and WT-AbLib-Omi groups, illustrating a marked increase in its relative frequency from 20.09% to 82.18% after Omicron panning.

We assessed the sequence diversity of VH and VL regions in WT-AbLib and Omi-AbLib after panning with different antigens (Figure 3A). WT-AbLib showed higher diversity when panned with WT antigens, with a normalized Shannon diversity of 0.605 for the VH region, and even higher diversity in the VL region. In contrast, after panning with BF.7 or XBB.1.5, WT-AbLib exhibited a significant decrease in VH diversity, with normalized Shannon diversity values of −0.205 and −0.272, reflecting extreme clonal dominance. Following BF.7 or XBB.1.5 enrichment, Omi-AbLib maintained higher diversity in both VH and VL regions, with minimal impact on light chain diversity.

At the repertoire level, enrichment of WT-AbLib with the SARS-CoV-2 WT antigen resulted in preferential usage of VH V–J gene pairings dominated by IGHV3-9/IGHJ4, IGHV1-46/IGHJ3, and IGHV3-66/IGHJ4 (Figure S1). To enable a direct comparison under Omicron-driven selection pressure, both WT-AbLib and Omi-AbLib were further enriched using the Omicron antigen, and the resulting VH usage profiles are shown in Figure 3B. Following Omicron enrichment, WT-AbLib exhibited a pronounced shift toward IGHV1-46/IGHJ3, whereas Omi-AbLib displayed distinct VH preferences, predominantly involving IGHV3-15, IGHV4-4, IGHV4-34, and IGHV3-66. Analysis of the VL region after Omicron enrichment revealed that WT-AbLib retained a relatively rich and evenly distributed light-chain family usage, whereas Omi-AbLib showed a more focused pattern, with enrichment of IGKV1-39, IGKV3-20, and IGKV1-5 (Figure 3B).

Given the similar results observed for BF.7 and XBB.1.5, we combined these into an Omicron group for further analysis of VH CDR3 sequence distribution (Figure 3C). The results revealed that the VH CDR3 sequences in WT-AbLib-WT were highly concentrated, with the top 8 sequences accounting for 82.14% of the total, and the dominant sequence comprising 22.77%. Under Omicron selection pressure, the VH CDR3 sequences in WT-AbLib-Omi became even more restricted, with the top 8 sequences representing 98.68%, of which the dominant sequence constituted 82.18%. In contrast, the sequence distribution in Omi-AbLib-Omi was more even, with the top 8 sequences cumulatively accounting for 68.11%. Additionally, the presence of identical heavy chain sequences in WT-AbLib-WT and WT-AbLib-Omi (20.09% and 82.18%, respectively) suggests that broadly binding antibodies originally induced by WT may be preferentially recalled by the immune system during the transition to Omicron, yet these antibodies lack neutralizing activity.

### 3.4. Functional Analysis and Binding Site Prediction of Dominant Antibodies

To investigate the functionality of high-frequency VH CDR3 sequences, we selected the top 6 heavy chains from WT-AbLib-WT, WT-AbLib-Omi, and Omi-AbLib-Omi groups (Figure 3C), paired them with their corresponding high-frequency light chains, and expressed them as IgG antibodies for ELISA binding and pseudovirus neutralization assays. The results indicated that antibodies sharing the same heavy chain but different light chains exhibited similar binding and neutralization activities, suggesting a dominant role of the heavy chain in determining antibody function. However, significant differences were observed among antibodies derived from different heavy chains. In the WT-AbLib-WT group, heavy chains ranked 1, 3, 4, and 5 displayed neutralizing activity, with WT-AbLib-WT-3 effectively neutralizing WT and BF.7, and further neutralizing XBB.1.5, demonstrating broad-spectrum neutralization capacity. Nevertheless, although WT-derived repertoires could give rise to antibodies capable of binding Omicron antigens, most high-frequency antibodies identified after Omicron panning from WT-AbLib-Omi and Omi-AbLib-Omi exhibited low binding affinity and weak neutralization activity in pseudovirus assays (Table 1). This indicates that clonal expansion during Omicron-driven selection does not necessarily correlate with neutralization potency.

**Figure 4:**
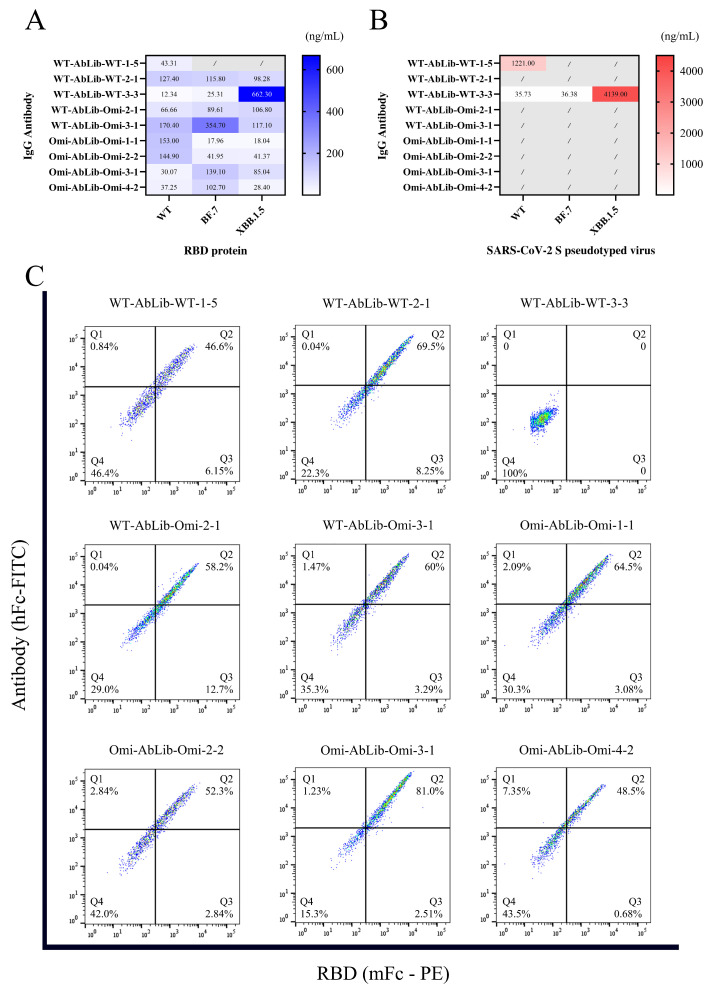
Functional characterization of dominant IgG antibodies. (**A**) Binding activities of IgG antibodies against the RBD proteins of SARS-CoV-2 WT, BF.7, and XBB.1.5, measured by ELISA. Results are shown as EC_50_ values (ng/mL), with lighter blue shade indicating stronger binding. Gray squares denote antibodies with no detectable binding at concentrations up to 10,000 ng/mL. (**B**) Neutralization activities of IgG antibodies against pseudoviruses bearing SARS-CoV-2 spike proteins (WT, BF.7, and XBB.1.5). IC_50_ values (ng/mL) are shown, with lighter red shade indicating stronger neutralizing activity. Gray squares indicate no detectable neutralization at concentrations up to 10,000 ng/mL. (**C**) Flow cytometry-based competition assay evaluating inhibition of RBD–hACE2 interaction. The x-axis indicates RBD (mFc-PE) fluorescence, and the y-axis indicates antibody (hFc-FITC) fluorescence. Quadrants represent distinct binding or blocking states, with percentages indicating the proportion of cells in each quadrant.

Further functional experiments demonstrated that antibodies from WT-AbLib-WT, WT-AbLib-Omi, and Omi-AbLib-Omi exhibited measurable and reproducible binding activity to both WT and Omicron antigens. Specifically, WT-AbLib-WT-1-5 and WT-AbLib-WT-3-3 antibodies showed strong binding to WT, with reduced activity against BF.7 and XBB.1.5, whereas Omi-AbLib-Omi-1-1, Omi-AbLib-Omi-2-2, and Omi-AbLib-Omi-4-2 antibodies displayed higher binding activity to Omicron than to WT (Figure 4A). However, in pseudovirus neutralization assays, WT-AbLib-WT-1-5 and WT-AbLib-WT-3-3 exhibited moderate neutralization against WT, with the latter also neutralizing BF.7 and XBB.1.5. In contrast, no detectable neutralization activity was observed within the tested concentration range for antibodies from WT-AbLib-Omi and Omi-AbLib-Omi groups (Figure 4B).

To assess the blocking efficacy of IgG antibodies against SARS-CoV-2 RBD, we evaluated their ability to inhibit RBD binding to hACE2 using flow cytometry (Figure 4C). The results showed that only WT-AbLib-WT-3-3 effectively blocked RBD-hACE2 interaction, consistent with its pseudovirus neutralization profile. Although WT-AbLib-WT-1-5 neutralized pseudovirus, it failed to block RBD-hACE2 binding, suggesting a neutralization mechanism that may be independent of direct receptor blockade. In the Omi-AbLib-Omi group, most cells clustered in the Q2 quadrant, indicating that although these antibodies retained RBD-binding capacity, they exhibited minimal inhibition of the RBD–hACE2 interaction, consistent with their lack of neutralizing activity.

Based on the AlphaFold3-simulated antigen–antibody complex structures (Table 2), the WT-AbLib-WT-3-3 antibody was predicted to target the RBM region of RBD, with substantial overlap with the hACE2 binding interface, thereby classifying it as a Class 1 antibody. Although the WT-AbLib-WT-1-5 antibody did not directly block RBD binding to hACE2, it may exert neutralizing activity by constraining conformational transitions between the “up” and “down” states of RBD. Other antibodies primarily bind to the lateral side of RBD, with no significant overlap with the hACE2 binding site, a region that is relatively conserved in Omicron.

## 4. Discussion

Immune memory plays a pivotal role in long-term pathogen defense, with prior studies showing that WT SARS-CoV-2-induced memory responses generate cross-reactive antibodies capable of reducing severe disease risk [19,20]. However, this memory can, under certain conditions, hinder adaptive responses to new variants through immune imprinting [9]. The receptor-binding domain (RBD) is critical for SARS-CoV-2 entry, with the receptor-binding motif (RBM) being the primary target of potent neutralizing antibodies [21,22,23]. Yet, immune imprinting and Omicron’s extensive Spike mutations [24,25,26] redirect antibody targeting from the RBM to conserved lateral RBD sites, favoring broadly binding but non-neutralizing antibodies. To investigate this dynamic, we employed phage-display technology to systematically analyze the screening characteristics and functional properties of antibody libraries—WT-AbLib (early 2020 WT convalescents) and Omi-AbLib (early 2023 Omicron convalescents)—revealing immune imprinting’s profound impact on antibody repertoires.

A key finding of this study is the dominance of broadly non-neutralizing antibodies following Omicron antigen enrichment, a phenomenon clearly elucidated through phage-display screening. In WT-AbLib, antibody diversity declined markedly following Omicron antigen enrichment (WT-AbLib-Omi). Specifically, dominant antibodies shifted from neutralizing IGHV3-66-derived clones targeting the RBM (12.95% in WT-AbLib-WT to <0.66% in WT-AbLib-Omi) to non-neutralizing IGHV1-46-derived clones targeting conserved lateral RBD sites (20.09% to 82.18%). 

In contrast, Omi-AbLib maintained higher diversity, yet its top 68.11% of heavy chains similarly favored non-neutralizing antibodies targeting the same conserved region. This shift aligns with reduced neutralizing antibody proportions post-Omicron infection reported elsewhere [11], supporting a role for immune imprinting in driving cross-reactive, non-neutralizing responses over variant-specific neutralization.

The prominence of non-neutralizing antibodies can be attributed to two synergistic mechanisms. First, frequent RBM mutations (e.g., K417N, E484A) [27,28] disrupt the binding of neutralizing antibodies, diminishing their enrichment efficiency in phage screening and efficacy against Omicron. Second, memory B cells, primed by WT exposure, preferentially recognize conserved RBD epitopes, leading to clonal competition that suppresses de novo B cell responses to Omicron-specific epitopes [25]. Together, these processes act synergistically during Omicron-driven selection. This dual mechanism explains the observed epitope transition from RBM to lateral RBD sites, a pattern less evident in prior in vivo antibody profiling studies [8]. Unlike single-cell sequencing or monoclonal antibody isolation, phage-display screening simulates immune selection in vitro, offering a controlled platform to quantify repertoire shifts and uncover antibodies overlooked by in vivo methods due to sampling or expression biases. For instance, the high prevalence of IGHV1-46 antibodies in WT-AbLib-Omi (82.18%) contrasts with their lower detection in plasma-derived analyses [11], highlighting phage technology’s sensitivity to conserved, non-neutralizing clones that may be underrepresented in plasma-derived analyses.

Functionally, WT-AbLib-WT contained neutralizing antibodies (e.g., WT-AbLib-WT-3) with broad-spectrum activity against WT, BF.7, and XBB.1.5, yet these were outcompeted under Omicron pressure. Conversely, IGHV1-46-derived antibodies, dominant in both WT-AbLib-Omi and Omi-AbLib-Omi, bound broadly but lacked neutralization, consistent with their targeting of a conserved lateral RBD site with minimal hACE2 overlap [29]. This site’s relatively lower mutation frequency in Omicron [30,31] suggests evolutionary stability. Although these antibodies are non-neutralizing, their conserved nature positions them as potential targets for antibody–drug conjugates, enabling the targeted delivery of antiviral agents. While immune imprinting delays Omicron-specific neutralizing responses, clinical data indicate prior exposure reduces reinfection severity [32]. Repeated Omicron exposures have been shown to induce broadly neutralizing antibodies [33,34,35], suggesting strategies to mitigate imprinting effects.

Importantly, the predominance of non-neutralizing antibodies does not imply a lack of immune protection. Accumulating evidence indicates that non-neutralizing antibodies can contribute to antiviral immunity through Fc-mediated effector functions, including antibody-dependent cellular cytotoxicity (ADCC), antibody-dependent cellular phagocytosis (ADCP), and complement activation, which may help to clear infected cells or virus complexes independently of direct neutralization [36] and in some experimental models with SARS-CoV-2 the presence of Fc-effector functionality has been associated with enhanced protection in vivo [37]. Moreover, real-world epidemiological data consistently show that individuals with prior infection and/or vaccination history experience reduced disease severity upon reinfection, even in the face of waning sterilizing immunity, including after studies of protection against symptomatic reinfection and breakthrough infections in large population cohorts [38]; this supports the concept that prior exposure shapes subsequent clinical outcomes. Therefore, immune imprinting should not be interpreted as immune failure, but rather as a redistribution of antibody specificities that may delay the generation of variant-specific neutralizing antibodies while still preserving partial protective immunity. In this framework, although the initial antibody response may be focused on conserved or previously encountered epitopes, it can nonetheless contribute to a reduced disease severity upon re-exposure or reinfection in the setting of complex immune memory and other effector mechanisms.

Despite its strengths, this study has limitations. The phage-display platform’s bias toward high-affinity binders may underrepresent rare neutralizing antibodies, necessitating complementary single-cell BCR sequencing or computational prediction [17,39]. Additionally, AlphaFold3-predicted structural models require experimental validation. Future work could explore in vivo validation of these findings to bridge in vitro and clinical contexts.

## 5. Conclusions

This study compared phage-display antibody libraries derived from early (WT-AbLib) and late (Omi-AbLib) COVID-19 convalescents, revealing that WT-AbLib exhibited a significant reduction in antibody diversity following Omicron antigen enrichment compared to WT antigen enrichment. The dominant antibodies shifted from potent neutralizing antibodies targeting the RBM to broadly non-neutralizing antibodies targeting conserved lateral sites on the RBD. In contrast, Omicron convalescents maintained higher antibody diversity after Omicron antigen enrichment, with dominant antibodies also being broadly non-neutralizing and targeting conserved RBD lateral sites. These findings elucidate the impact of pre-existing immunity on subsequent antibody repertoires during SARS-CoV-2 infection, providing an immunological explanation for the frequent recurrence of Omicron infections and robust immune evasion. Moreover, they offer critical scientific insights for the development of COVID-19 vaccines and therapeutic antibodies.

## Figures and Tables

**Table 1:** Sequences and functional characteristics of IgG antibodies derived from dominant heavy chains in WT-AbLib and Omi-AbLib panned with SARS-CoV-2 WT and Omicron variants.

Group	IgG	Heavy Chain			Light Chain			Elisa OD_450_				Anti-Pseudovirus		
		Proportion	VJ	CDR3	Proportion	VJ	CDR3	HuFab	WT	BF.7	XBB.1.5	WT	BF.7	XBB.1.5
WT-AbLib panning with WT	WT-AbLib-WT-1-1	22.77%	IGHV3-9*01/IGHJ4*01	AKDVYSESGSGSYYDY	8.33%	IGKV1-39*01/IGKJ5*01	QQTYSAPTT	2.98	3.20	0.06	0.05	58%	13%	5%
	WT-AbLib-WT-1-2	22.77%	IGHV3-9*01/IGHJ4*01	AKDVYSESGSGSYYDY	6.25%	IGLV1-51*01/IGLJ3*02	GTWDSSLSAWV	2.00	3.32	0.06	0.04	61%	11%	−14%
	WT-AbLib-WT-1-3	22.77%	IGHV3-9*01/IGHJ4*01	AKDVYSESGSGSYYDY	2.08%	IGKV3-20*02/IGKJ1*01	QQHGRT	3.48	4.21	0.06	0.05	51%	−4%	−11%
	WT-AbLib-WT-1-4	22.77%	IGHV3-9*01/IGHJ4*01	AKDVYSESGSGSYYDY	2.08%	IGKV1-27*01/IGKJ4*01	QKYNSAPLT	2.35	2.44	0.06	0.04	73%	23%	17%
	WT-AbLib-WT-1-5*	22.77%	IGHV3-9*01/IGHJ4*01	AKDVYSESGSGSYYDY	2.08%	IGLV6-57*01/IGLJ3*02	QSYDSSNQWV	2.97	3.88	0.06	0.05	100%	18%	14%
	WT-AbLib-WT-1-6	22.77%	IGHV3-9*01/IGHJ4*01	AKDVYSESGSGSYYDY	2.08%	IGLV2-14*03/IGLJ2*01	SSYTSSSTRG	2.92	4.62	0.06	0.05	68%	10%	7%
	WT-AbLib-WT-2-1*	20.09%	IGHV1-46*04/IGHJ3*02	ARDGGYIPAHDAFDI	15.91%	IGKV3-20*01/IGKJ4*01	QQYGSSLT	3.27	3.67	3.10	3.58	35%	5%	−9%
	WT-AbLib-WT-2-2	20.09%	IGHV1-46*04/IGHJ3*02	ARDGGYIPAHDAFDI	6.82%	IGKV1-39*01/IGKJ1*01	QQTYLVPNT	3.80	4.63	2.20	3.13	−7%	18%	−5%
	WT-AbLib-WT-2-3	20.09%	IGHV1-46*04/IGHJ3*02	ARDGGYIPAHDAFDI	4.55%	IGLV2-14*03/IGLJ1*01	NSYTTNSTYV	2.92	3.78	1.82	2.61	5%	12%	−5%
	WT-AbLib-WT-2-4	20.09%	IGHV1-46*04/IGHJ3*02	ARDGGYIPAHDAFDI	4.55%	IGLV6-57*03/IGLJ3*02	QSFDANFHWV	1.79	1.87	0.52	0.82	6%	20%	−9%
	WT-AbLib-WT-2-5	20.09%	IGHV1-46*04/IGHJ3*02	ARDGGYIPAHDAFDI	2.27%	IGKV3-20*02/IGKJ1*01	QQHGRT	3.05	3.12	1.54	2.04	−6%	6%	14%
	WT-AbLib-WT-2-6	20.09%	IGHV1-46*04/IGHJ3*02	ARDGGYIPAHDAFDI	2.27%	IGKV1-39*01/IGKJ3*01	QQSYSTLFT	2.93	2.86	1.01	1.82	13%	3%	8%
	WT-AbLib-WT-3-1	12.95%	IGHV3-66*01/IGHJ4*01	ARSYGDFYVDF	13.79%	IGKV1-9*01/IGKJ5*01	QHLNNYPIS	3.49	3.06	2.48	0.04	94%	82%	2%
	WT-AbLib-WT-3-2	12.95%	IGHV3-66*01/IGHJ4*01	ARSYGDFYVDF	10.34%	IGKV3-20*01/IGKJ4*01	QQYGSSLT	2.73	2.08	2.39	0.04	83%	84%	−13%
	WT-AbLib-WT-3-3*	12.95%	IGHV3-66*01/IGHJ4*01	ARSYGDFYVDF	10.34%	IGKV3-20*01/IGKJ1*01	QQHGRT	3.44	3.21	2.17	0.53	86%	87%	49%
	WT-AbLib-WT-3-4	12.95%	IGHV3-66*01/IGHJ4*01	ARSYGDFYVDF	6.90%	IGKV1-39*01/IGKJ5*01	QQTYSAPTT	1.57	1.57	2.74	0.06	64%	54%	3%
	WT-AbLib-WT-4-1	11.61%	IGHV3-9*01/IGHJ4*01	AKDVYSESASGSYYDY	16.00%	IGKV1-39*01/IGKJ5*01	QQTYSAPTT	2.38	2.59	0.06	0.04	55%	7%	12%
	WT-AbLib-WT-4-2	11.61%	IGHV3-9*01/IGHJ4*01	AKDVYSESASGSYYDY	12.00%	IGKV1-33*01/IGKJ3*01	QHYDNQPSFT	2.05	2.97	0.06	0.04	60%	19%	18%
	WT-AbLib-WT-4-3	11.61%	IGHV3-9*01/IGHJ4*01	AKDVYSESASGSYYDY	8.00%	IGKV3-20*02/IGKJ1*01	QQHGRT	2.05	3.64	0.05	0.04	76%	16%	8%
	WT-AbLib-WT-4-4	11.61%	IGHV3-9*01/IGHJ4*01	AKDVYSESASGSYYDY	3.45%	IGLV1-40*01/IGLJ3*02	QTFDSSLRAWV	2.21	3.99	0.06	0.04	67%	28%	19%
	WT-AbLib-WT-4-5	11.61%	IGHV3-9*01/IGHJ4*01	AKDVYSESASGSYYDY	4.00%	IGKV1-39*01/IGKJ2*01	QQSSSTPQT	3.07	3.11	0.05	0.04	75%	18%	18%
	WT-AbLib-WT-4-6	11.61%	IGHV3-9*01/IGHJ4*01	AKDVYSESASGSYYDY	4.00%	IGKV3-20*01/IGKJ1*01	QQYGSSPPWT	3.65	3.66	0.05	0.04	50%	9%	13%
	WT-AbLib-WT-5-1	5.36%	IGHV3-66*01/IGHJ6*01	ARDLGPRGMDV	41.67%	IGKV1-33*01/IGKJ3*01	QHYDNQPSFT	2.26	3.22	0.06	0.04	77%	22%	15%
	WT-AbLib-WT-5-2	5.36%	IGHV3-66*01/IGHJ6*01	ARDLGPRGMDV	8.33%	IGKV3-20*01/IGKJ1*01	QQYGSSPPWT	3.09	2.90	0.05	0.04	61%	26%	11%
	WT-AbLib-WT-6-1	4.91%	IGHV3-64D*06/IGHJ4*01	VKDSPVVTAMVTVFDY	100.00%	IGKV1-39*01/IGKJ3*01	QQSYSTLFT	1.92	2.09	0.53	0.76	−12%	12%	5%
WT-AbLib panning with Omicron	WT-AbLib-Omi-1-1*	82.19%	IGHV1-46*04/IGHJ3*02	ARDGGYIPAHDAFDI	38.73%	IGKV3-20*01/IGKJ4*01	QQYGSSLT	3.09	2.86	3.15	4.05	17%	−9%	−10%
	WT-AbLib-Omi-1-2	82.19%	IGHV1-46*04/IGHJ3*02	ARDGGYIPAHDAFDI	13.62%	IGKV1-39*01/IGKJ4*01	QQSYSTPLT	3.62	3.31	2.63	3.23	9%	−12%	−9%
	WT-AbLib-Omi-1-3	82.19%	IGHV1-46*04/IGHJ3*02	ARDGGYIPAHDAFDI	10.21%	IGKV1-6*01/IGKJ1*01	LQDYNYPWT	3.51	4.17	2.31	2.38	−10%	−8%	−8%
	WT-AbLib-Omi-1-4	82.19%	IGHV1-46*04/IGHJ3*02	ARDGGYIPAHDAFDI	2.55%	IGKV3-20*01/IGKJ4*01	QQYGTPPLT	2.78	2.55	1.50	2.94	−4%	−10%	−6%
	WT-AbLib-Omi-1-5	82.19%	IGHV1-46*04/IGHJ3*02	ARDGGYIPAHDAFDI	0.85%	IGKV1-9*01/IGKJ4*01	QQLNSYPLT	2.89	3.23	3.20	3.42	−4%	−9%	3%
	WT-AbLib-Omi-1-6	82.19%	IGHV1-46*04/IGHJ3*02	ARDGGYIPAHDAFDI	0.85%	IGKV3-20*01/IGKJ1*01	QQYGSSRT	3.58	4.18	4.66	4.48	−7%	−14%	−8%
	WT-AbLib-Omi-1-7	82.19%	IGHV1-46*04/IGHJ3*02	ARDGGYIPAHDAFDI	0.43%	IGLV2-14*03/IGLJ2*01	SSYSTSSPVV	3.39	3.76	4.69	4.18	9%	−19%	−3%
	WT-AbLib-Omi-2-1*	6.93%	IGHV3-30*18/IGHJ4*01	AKVAGPYCSAGNCYGGDFDY	75.00%	IGLV6-57*03/IGLJ2*01	QSYNKTNLV	4.16	4.30	4.05	4.23	10%	−12%	−6%
	WT-AbLib-Omi-3-1*	3.96%	IGHV3-30*18/IGHJ4*01	AKAGGPYCGGSSCYGRFFDY	64.33%	IGKV1-33*01/IGKJ5*01	QQYGNLPIT	2.40	2.20	2.46	2.30	−8%	−11%	−19%
	WT-AbLib-Omi-3-2	3.96%	IGHV3-30*18/IGHJ4*01	AKAGGPYCGGSSCYGRFFDY	35.71%	IGKV1-33*01/IGKJ4*01	QQFHNVPLT	1.76	1.78	1.88	1.64	7%	−17%	−4%
	WT-AbLib-Omi-4-1	2.64%	IGHV1-3*04/IGHJ4*01	ARAIYGPGSPPIY	15.79%	IGLV6-57*01/IGLJ3*02	QSYDSNNPWV	2.77	2.65	0.93	0.80	14%	−8%	−6%
	WT-AbLib-Omi-5-1	0.99%	IGHV5-51*01/IGHJ6*01	ARHVSPYYYGTGSYSGGMDV	87.50%	IGKV1-27*01/IGKJ4*01	QKYDSAPLT	3.40	4.39	2.66	3.26	10%	−13%	−9%
	WT-AbLib-Omi-6-1	0.66%	IGHV3-30*01/IGHJ6*01	VLGSFYDFFTGYRGLDF	100.00%	IGKV2-28*01/IGKJ3*01	MQGVESVFS	3.43	1.22	0.10	0.06	4%	−10%	−15%
Omi-AbLib panning with Omicron	Omi-AbLib-Omi-1-1*	25.41%	IGHV4-34*12/IGHJ5*02	ARGLWIAGLRFDP	26.09%	IGKV1-39*01/IGKJ2*01	QQSYSTPYT	2.97	1.30	3.43	2.39	−10%	16%	−5%
	Omi-AbLib-Omi-1-2	25.41%	IGHV4-34*12/IGHJ5*02	ARGLWIAGLRFDP	21.74%	IGKV1-39*01/IGKJ2*01	QQSYSPPYT	2.74	1.20	3.03	2.25	−12%	23%	−10%
	Omi-AbLib-Omi-1-3	25.41%	IGHV4-34*12/IGHJ5*02	ARGLWIAGLRFDP	10.87%	IGKV1-39*01/IGKJ1*01	QQSYSTPRT	3.80	1.36	2.21	1.36	15%	20%	−13%
	Omi-AbLib-Omi-1-4	25.41%	IGHV4-34*12/IGHJ5*02	ARGLWIAGLRFDP	4.35%	IGKV1-39*01/IGKJ1*01	QQSYSTPVT	3.26	1.82	4.15	3.80	−8%	3%	−11%
	Omi-AbLib-Omi-2-1	12.97%	IGHV3-15*02/IGHJ4*01	TTDAAYTWSPDY	88.00%	IGKV1-39*01/IGKJ4*01	QQSYSTPLT	2.28	3.30	2.47	2.34	−12%	18%	−1%
	Omi-AbLib-Omi-2-2*	12.97%	IGHV3-15*02/IGHJ4*01	TTDAAYTWSPDY	42.86%	IGKV1-39*01/IGKJ1*01	QQSYSTPRT	3.80	3.78	5.23	4.80	−18%	14%	5%
	Omi-AbLib-Omi-2-3	12.97%	IGHV3-15*02/IGHJ4*01	TTDAAYTWSPDY	4.00%	IGKV1-39*01/IGKJ5*01	QQSYSSLIT	2.55	2.91	2.95	2.76	−13%	6%	−12%
	Omi-AbLib-Omi-2-4	12.97%	IGHV3-15*02/IGHJ4*01	TTDAAYTWSPDY	4.00%	IGKV1-39*01/IGKJ1*01	QQSYSTPWT	2.77	2.93	3.20	3.12	1%	7%	−12%
	Omi-AbLib-Omi-3-1*	8.11%	IGHV3-15*01/IGHJ4*01	TTDAGYTWSPDY	100.00%	IGKV1-39*01/IGKJ1*01	QQSYSTPWT	2.89	3.75	1.05	1.72	−7%	29%	−13%
	Omi-AbLib-Omi-4-1	4.86%	IGHV1-46*01/IGHJ3*02	ARLGSGIPAARDGLDI	55.56%	IGKV1-39*01/IGKJ4*01	QQSYSTPLT	1.71	1.67	1.42	1.67	−4%	17%	−2%
	Omi-AbLib-Omi-4-2*	4.86%	IGHV1-46*01/IGHJ3*02	ARLGSGIPAARDGLDI	11.11%	IGKV1-39*01/IGKJ1*01	QQSYSTPWT	3.63	4.47	3.78	3.92	−13%	19%	−6%
	Omi-AbLib-Omi-5-1	4.86%	IGHV4-4*02/IGHJ4*01	ARKKGWLRGFNDY	93.33%	IGLV6-57*02/IGLJ2*01	QSYDTGNQV	2.06	3.82	1.64	0.87	−12%	5%	6%
	Omi-AbLib-Omi-6-1	4.32%	IGHV3-15*07/IGHJ4*01	TTFSRPDH	25.00%	IGKV4-1*01/IGKJ2*01	QQYYSTPYT	2.54	2.70	4.12	3.00	−7%	18%	−16%
	Omi-AbLib-Omi-6-2	4.32%	IGHV3-15*07/IGHJ4*01	TTFSRPDH	12.50%	IGKV3-20*01/IGKJ2*01	QQYGSSPYT	3.12	3.59	3.32	2.97	−17%	21%	−18%

Notes: The table summarizes the characteristics of various VH-VL antibody combinations, including heavy and light chain VJ genes, CDR3 sequences, proportions, ELISA OD_450_ values, and pseudovirus neutralization activities. VH chains are ranked according to their frequency within the antibody libraries. Antibody nomenclature follows the format: Library-Enriched Variant–VH Frequency Rank–VL Frequency Rank. Both ELISA and pseudovirus neutralization assays were performed using Fab-containing supernatants filtered through a 0.22 µm membrane and diluted 1:10. Higher values indicate stronger antibody binding or neutralization activity. Alternating gray and white background colors are used to distinguish the three major antibody groups. Red shading indicates positive results, defined as ELISA OD_450_ > 0.13 or pseudovirus neutralization > 50%.

**Table 2:** Predicted binding sites of dominant IgG antibodies on RBD proteins of SARS-CoV-2 WT and Omicron variants.

IgG	WT		BF.7		XBB.1.5	
WT-AbLib-WT-1-5	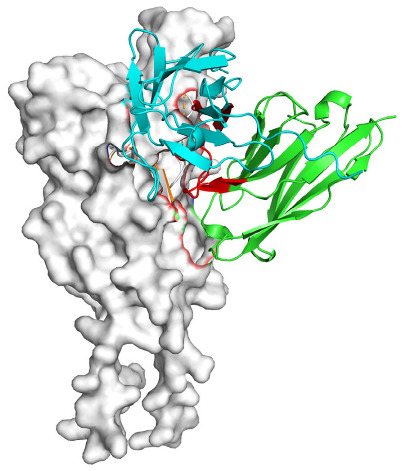	T345, R346, N354, R355, K356, R357, S359, N360, Y396, R466, I468, T470				
WT-AbLib-WT-2-1	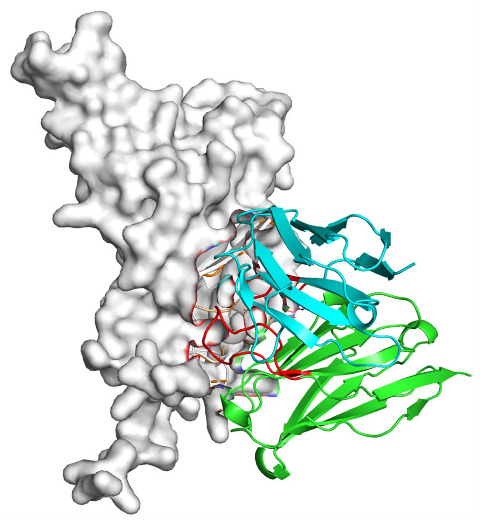	Y369, N370, S375, F377, K378, C379, Y380, G381, V382, S383, P384, T385, K386, N388, D389, L390, K528, T531, N536	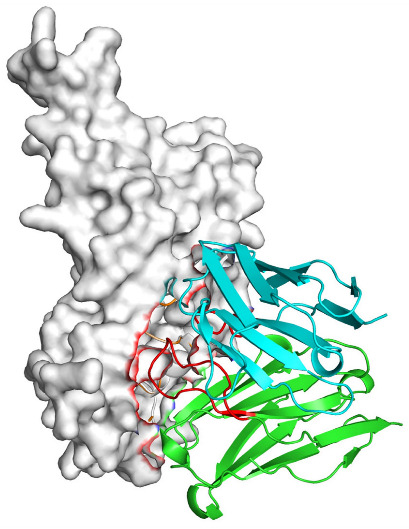	F375, F377, K378, C379, Y380, G381, V382, S383, P384, T385, K386, N388, D389, L390, K528, V534, V539	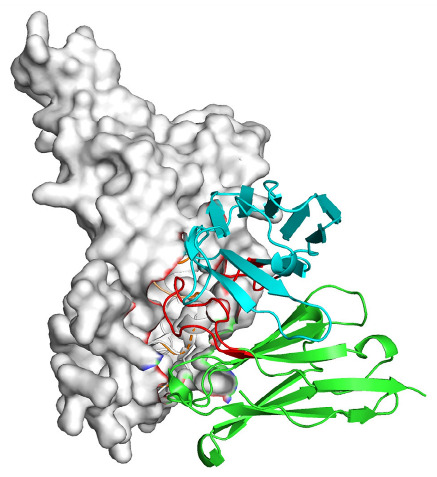	K378, C379, Y380, G381, V382, S383, P384, T385, K386, D389, L390, K528, S530, N532
WT-AbLib-WT-3-3	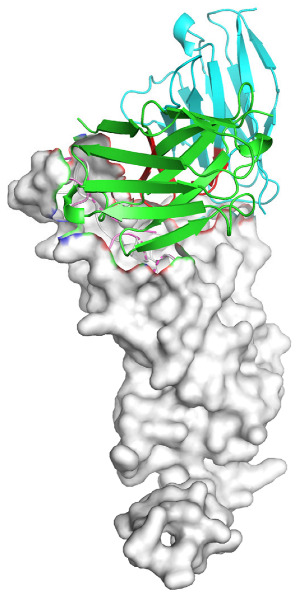	R403, T415, G416, K417, D420, Y421, Y453, L455, F456, R457, K458, N460, Y473, Q474, A475, G476, S477, F486, N487, Y489, Q493, T500, G502, Y505	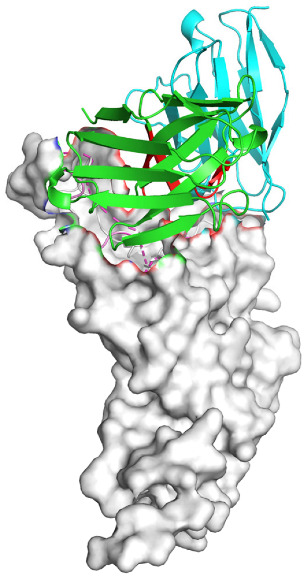	R403, T415, G416, N417, D420, Y421, Y453, L455, F456, R457, K458, N460, Y473, Q474, A475, G476, N477, F486, N487, Y489, Q493, T500, Y501, G502, H505	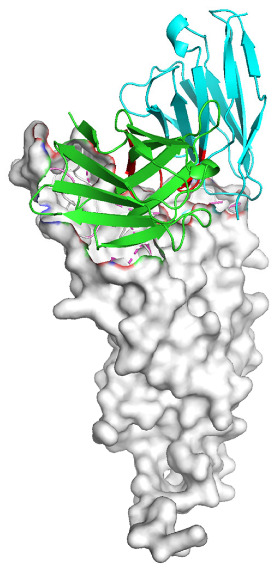	R403, T415, G416, N417, D420, Y421, Y453, L455, F456, R457, K458, K460, Y473, Q474, A475, G476, N477, K478, N487, Y489, Q493, T500, Y501, G502, H505
WT-AbLib-Omi-2-1	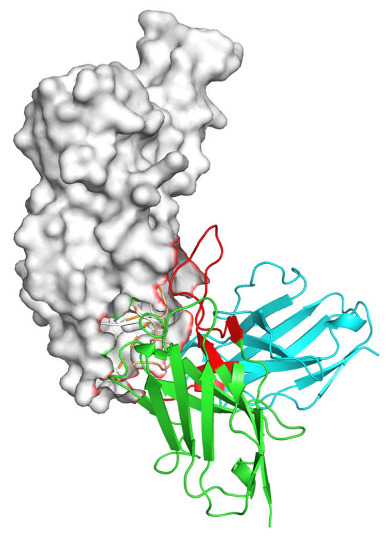	E324, V327, R328, F329, P330, N331, I332, T333, N334, L335, P337, E340, R357, I358, S359, N360, C361, V362, A520, P521, T523, S530	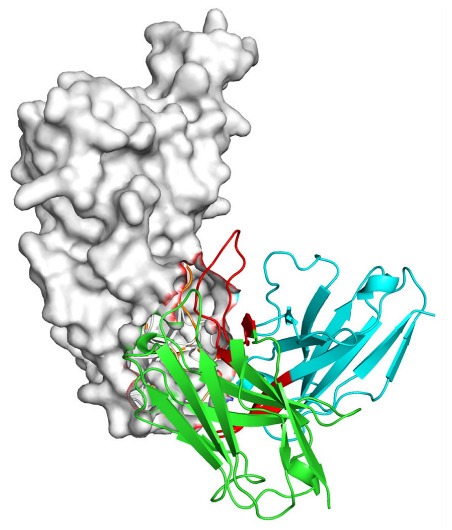	R328, F329, P330, N331, I332, T333, N334, L335, P337, E340, K356, R357, I358, S359, N360, V362, T393, A520, P521, T523, K529	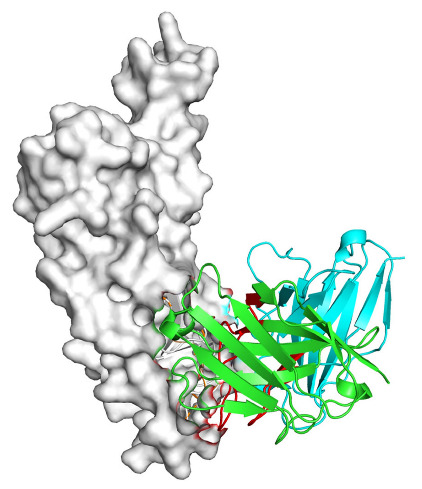	R328, F329, N331, I332, T333, N334, L335, P337, H339, E340, R357, S359, N360, P521, A522, L533, V534, K535
WT-AbLib-Omi-3-1	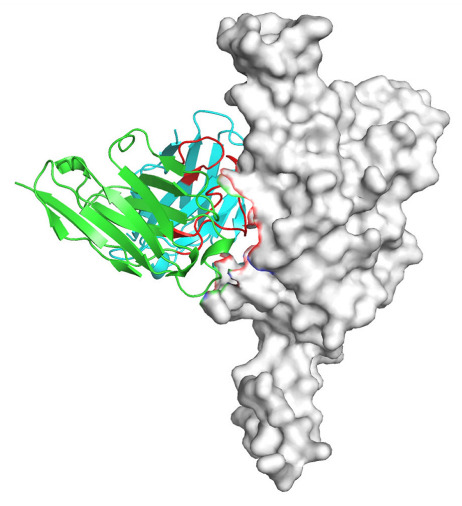	R355, R357, S359, N360, Y396, P426, D428, K462, P463, F464, E465, R466, E516, L518, H519, T523	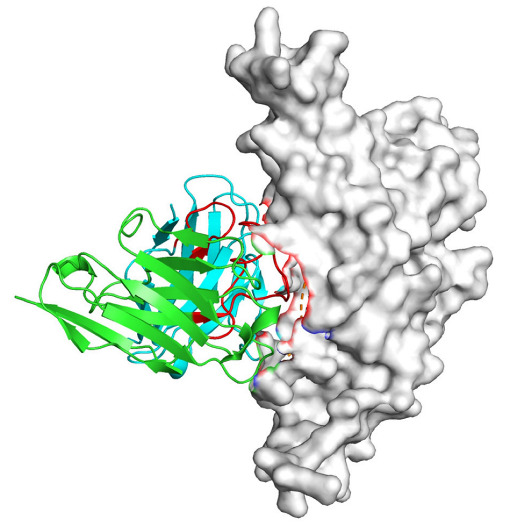	R355, R357, S359, N360, Y396, P426, D428, K462, P463, F464, E465, R466, I468, E516, L518, H519, T523	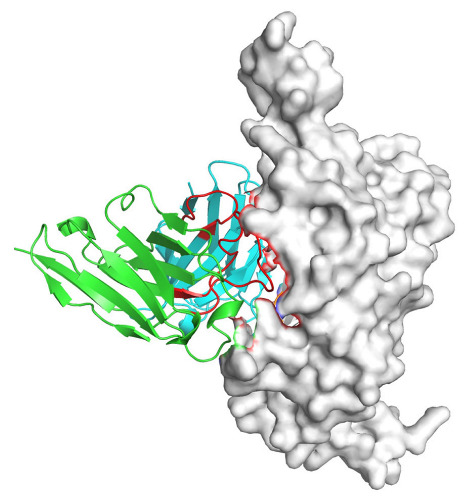	A352, W353, N354, R355, K356, R357, S359, N394, Y396, D428, K462, P463, F464, E465, R466, I468, F515, E516, L518, H519, A520
Omi-AbLib-Omi-1-1	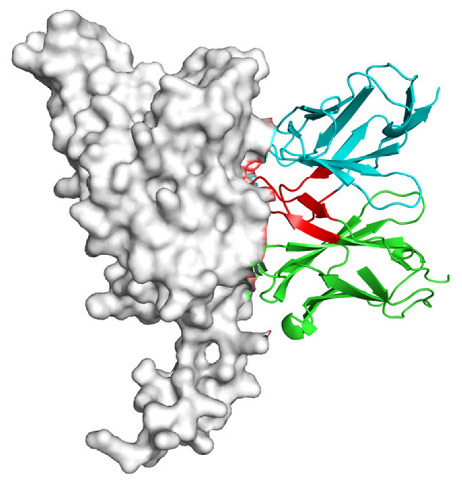	L335, F342, N343, A344, T345, S366, V367, L368, N370, S371, S373, F374, N440, L441	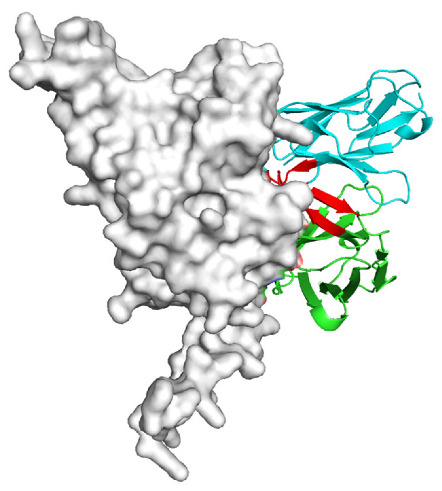	T333, L335, D339, F342, N343, A344, T345, V362, D364, S366, V367, L368, N370, F371, F375, P527	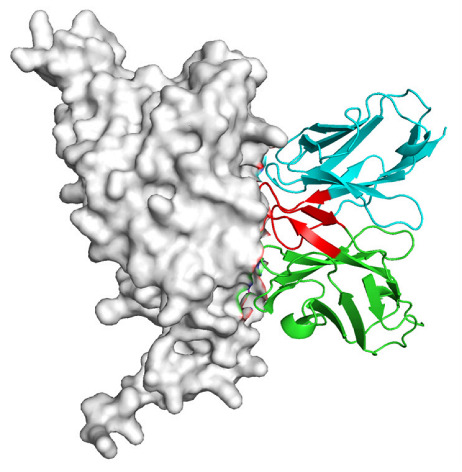	T333, N334, L335, F342, N343, A344, T345, V362, D364, S366, V367, I368, N370, F371, P527
Omi-AbLib-Omi-2-2	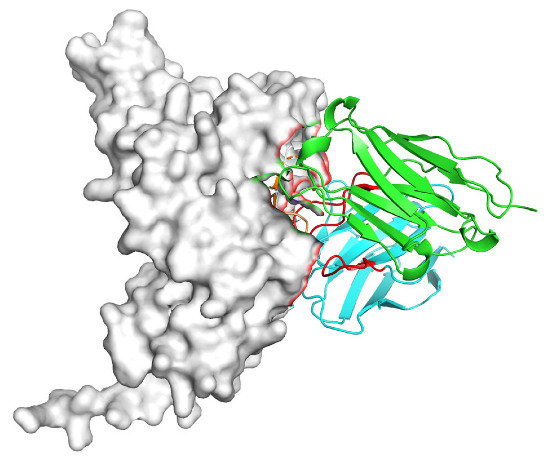	G339, F342, N343, S366, V367, L368, S371, A372, S373, F374, S375, W436, N437, N440, V503, Q506, Y508	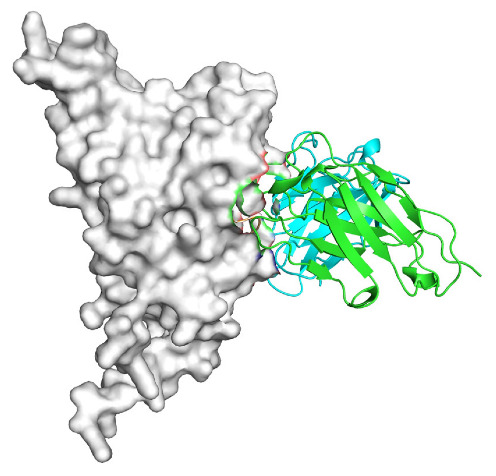	L335, D339, N343, D364, V367, N370, F371, A372, P373, F374, F375, W436, N437, K440, L441, Q506	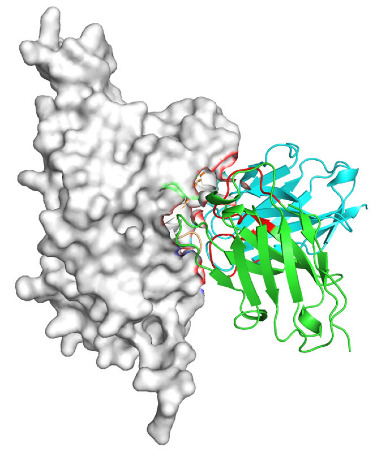	N343, N370, F371, A372, P373, F374, F375, W436, N437, N439, K440, L441, P499, V503, Q506, Y508
Omi-AbLib-Omi-3-1	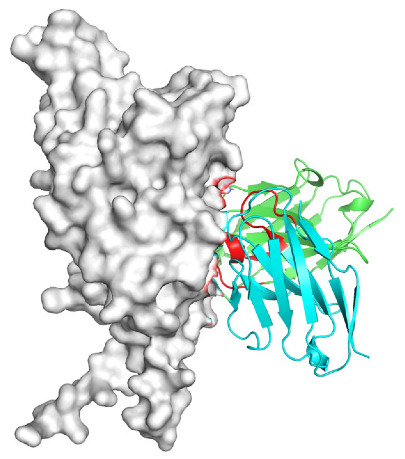	L335, C336, P337, G339, E340, F342, N343, A344, T345, D364, V367, S371, A372, S373, L441	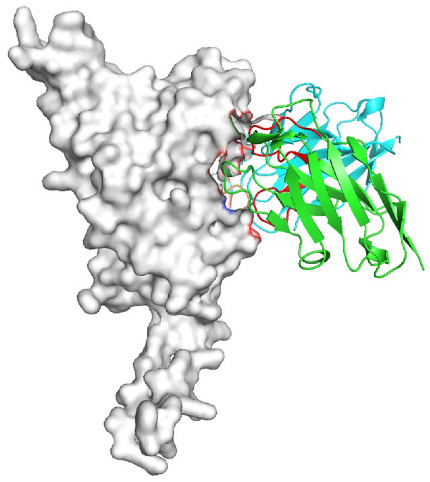	F342, N343, N370, F371, A372, P373, F374, F375, W436, N437, N439, K440, L441, V503, Q506	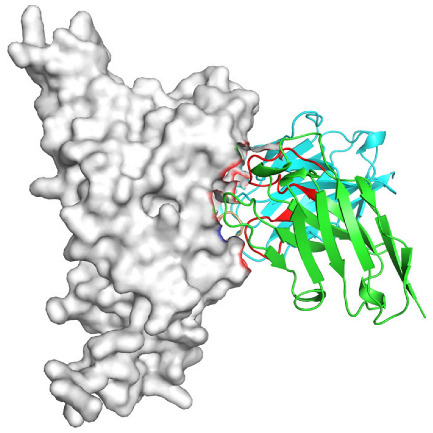	F342, N343, A344, N370, F371, A372, P373, F374, F375, W436, N437, K440, L441, V503, Q506
Omi-AbLib-Omi-4-2	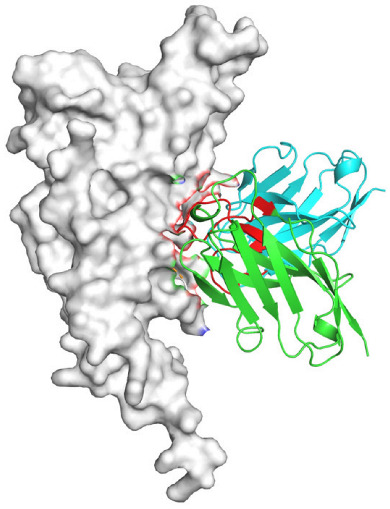	R355, R357, T393, N394, Y396, D428, K462, P463, F464, E465, S514, E516, L518, H519, A520	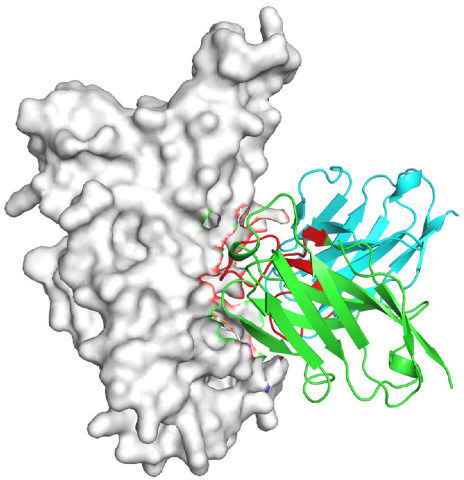	R319, R355, R357, N394, Y396, D428, K462, P463, F464, E465, S514, E516, L518, H519, A520	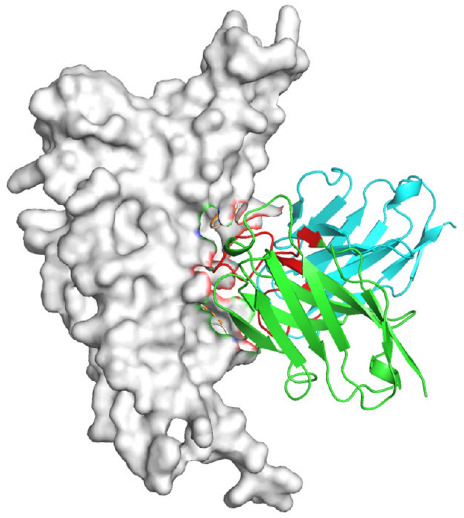	R355, R357, N394, Y396, D428, K462, P463, F464, E465, S514, E516, L518, H519, A520

Notes: This table lists the predicted binding sites and docking models of IgG antibodies identified from the WT-AbLib and Omi-AbLib libraries against the RBD proteins of SARS-CoV-2 (WT, BF.7, and XBB.1.5). Neutralizing antibodies are highlighted in light gray. In the models, the RBD is depicted as a gray surface, the antibody heavy chain (VH) in green, and the light chain (VL) in blue, with the antibody CDR3 regions highlighted in red. The binding sites presented in the table were derived from molecular docking simulations, with key residues annotated by their amino acid positions.

## Data Availability

All data generated or analyzed during this study are included in this published article.

## Supplementary Materials

The following supporting information can be downloaded at: https://www.mdpi.com/article/10.3390/v18010132/s1, Figure S1: VJ gene pairwise preferences in WT-AbLib panned with the SARS-CoV-2 WT.
