# Supplementary material for: Immune Imprinting Identified in Phage-Display Antibody Libraries Derived from Early Wild-Type and Late Omicron COVID-19 Convalescents
Source: Viruses. 2026 Jan 20;18(1):132. doi: 10.3390/v18010132 (PMC12846543; doi:10.3390/v18010132)
Supplement: Supplementary file 1 [file viruses-18-00132-s001.zip › viruses-4075010-supplementary.pdf]

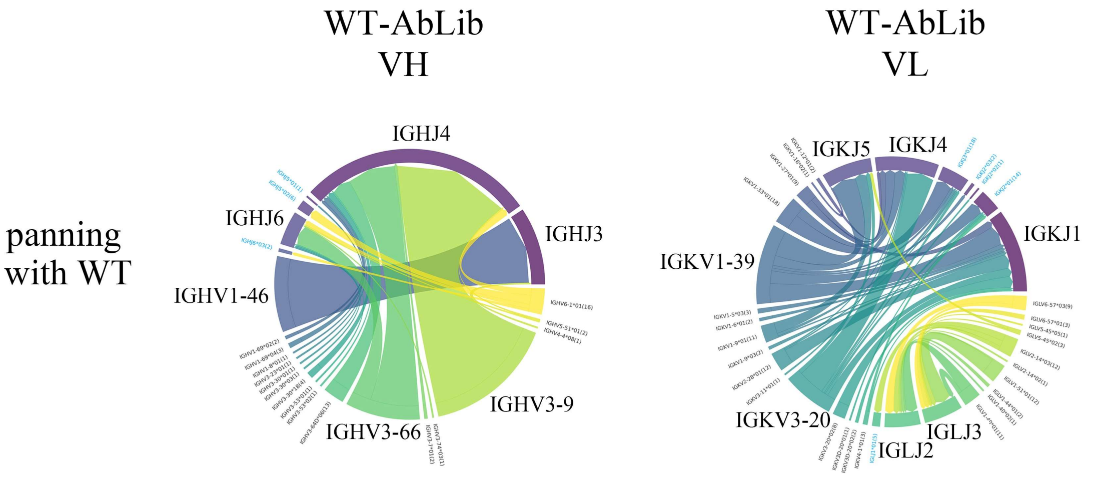

Figure S1. VJ gene pairwise preferences in WT-AbLib panned with the SARS-CoV-2 WT. Chord diagrams illustrate the usage of VH (**left**) and VL (**right**) gene families in the WT-AbLib library following panning with the WT. Arc length represents the relative abundance of each gene family, while connecting ribbons indicate enriched V–J pairings. Colors correspond to distinct VH or VL gene families.
